# The 3' to 5' Exoribonuclease DIS3: From Structure and Mechanisms to Biological Functions and Role in Human Disease

**DOI:** 10.3390/biom5031515

**Published:** 2015-07-17

**Authors:** Sophie R. Robinson, Antony W. Oliver, Timothy J. Chevassut, Sarah F. Newbury

**Affiliations:** 1 Medical Research Building, Brighton and Sussex Medical School, University of Sussex, Falmer, Brighton BN1 9PS, UK; E-Mails: s.robinson2@bsms.ac.uk (S.R.R.); t.chevassut@bsms.ac.uk (T.J.C.); 2 Genome Damage and Stability Centre, School of Life Sciences, University of Sussex, Falmer, Brighton BN1 9RQ, UK; E-Mail: antony.oliver@sussex.ac.uk

**Keywords:** DIS3, exoribonuclease, multiple myeloma, RNA stability

## Abstract

DIS3 is a conserved exoribonuclease and catalytic subunit of the exosome, a protein complex involved in the 3' to 5' degradation and processing of both nuclear and cytoplasmic RNA species. Recently, aberrant expression of DIS3 has been found to be implicated in a range of different cancers. Perhaps most striking is the finding that DIS3 is recurrently mutated in 11% of multiple myeloma patients. Much work has been done to elucidate the structural and biochemical characteristics of DIS3, including the mechanistic details of its role as an effector of RNA decay pathways. Nevertheless, we do not understand how DIS3 mutations can lead to cancer. There are a number of studies that pertain to the function of DIS3 at the organismal level. Mutant phenotypes in *S. pombe*, *S. cerevisiae* and *Drosophila* suggest DIS3 homologues have a common role in cell-cycle progression and microtubule assembly. DIS3 has also recently been implicated in antibody diversification of mouse B-cells. This article aims to review current knowledge of the structure, mechanisms and functions of DIS3 as well as highlighting the genetic patterns observed within myeloma patients, in order to yield insight into the putative role of DIS3 mutations in oncogenesis.

## 1. Introduction

A fine balance must be achieved between the synthesis and degradation of RNAs in the cell. Mutations that cause defects in RNA turnover can have significant consequences on cellular function [1,2,3,4]. Transcriptional control provides one means by which to regulate gene expression but post-transcriptional gene regulation through RNA degradation is also critical. RNA degradation involves a number of complex and interconnected pathways that all converge on common mechanisms of decay, through the recruitment of ribonucleases. As well as functioning in RNA turnover as a means of gene regulation, ribonucleases also function to remove aberrant mRNAs to prevent accumulation of toxic protein products. Moreover, the majority of primary transcripts are subject to exo or endonucleolytic processing by ribonucleases to produce mature RNAs that have diverse functions within the cell.

DIS3 is a highly conserved 3' to 5' exoribonuclease that provides catalytic activity to a multi-subunit complex, the exosome [5]. A large number of studies have elucidated the biochemical and structural characteristics of DIS3 as well as its mechanism of action in various RNA processing pathways. DIS3 has a diverse range of functions within RNA metabolism including mRNA quality control [6,7] regulation of gene expression [8,9] and small RNA processing [10,11]. Studies using mutant phenotypes have also revealed functions of DIS3 at the organismal level such as chromosome segregation [12,13], cell-cycle progression [14,15], spindle assembly [16] and even the diversification of antibodies in B-cells [17]. Gene expression profiling studies have shown aberrant expression of DIS3 in a small range of different cancers [18,19,20,21,22,23,24] and recently whole genome/whole exome and amplicon sequencing studies have revealed DIS3 to be recurrently mutated in multiple myeloma [25,26,27,28,29]. However, whether DIS3 is an oncogene or tumour-suppressor gene remains to be proven by functional investigations. Additionally, more genome-sequencing studies may help reveal patterns of mutation exclusivity and/or cooperation thus enhancing functional insight into the role of DIS3 mutations in oncogenesis.

This article aims to review the current knowledge of DIS3. We will discuss the structural and catalytic properties of DIS3, as well as its molecular and biological functions, role in disease and common genetic patterns observed in patients, in order to provide a basis on which to investigate the role of DIS3 in cancer progression.

## 2. Conservation, Structure, Mechanistic Functions and Sub-Cellular Localisation of DIS3

**Figure 1:**
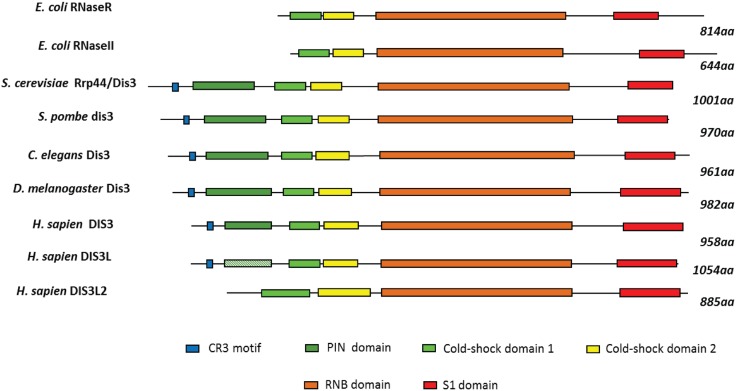
Domain organisation of members of the RNR/RNase II superfamily. Members of this family have a similar modular domain organization. The N-terminal region is variable but cold-shock domain 1 and cold-shock domain 2 are present in all members, followed by a RNB domain and an S1 domain. At the N-terminus, *S. cerevisiae* Rrp44/Dis3 and some other members also contain a conserved CR3 motif and a catalytic PIN domain. However, mutations in human DIS3L render the PIN domain inactive (hatched). Dis3L2 has lost the N-terminal extension but contains extended cold shock domains. See text for details of domain functions. See reference [31] for more detail on domain compositions.

DIS3 was first discovered in *S. pombe* mutants that were *defective in sister chromatid disjoining* [12]. Orthologues of DIS3 belong to the RNase II/RNR superfamily and exist in most organisms from bacteria to humans [30]. Members of this family show very high sequence conservation as well as functional conservation as demonstrated by the genetic complementation of mutant yeast Dis3 (Rrp44) with the human homologue [10,11]. Some eukaryotes have more than one homologue. The domain architecture differs slightly between homologues (Figure 1, see [31] for more detail on domain composition). Human DIS3 has an exonucleolytic RNB domain, two cold shock domains (CSDS) and an S1 domain which non-specifically bind RNA and an endonucleolytic PIN domain [31,32,33]. At the N-terminus a CR3 motif consisting of three cysteine residues, has an important structural role [34]. Humans also contain two further homologues, DIS3L and DIS3L2, which differ in the presence or absence of the PIN domain [31,35]. DIS3L does possess a PIN domain but it is rendered inactive by mutations in two important acidic residues; whereas the PIN domain is completely absent from DIS3L2. Recent evidence suggests that DIS3L2 is a paralogue of DIS3 which functions in different pathways, independent from the exosome [36,37,38].

Structural analyses and RNase protection experiments have revealed a common model for the mechanism of action of DIS3. DIS3 is a highly processive, hydrolytic enzyme with a preference for substrates with phosphorylated 5' termini [39]. It hydrolyses single-stranded RNA in a 3' to 5' direction, releasing one nucleotide at a time and leaving a product a few nucleotides long [40]. Exonuclease activity is dependent on four conserved aspartic acid residues that coordinate two magnesium ions in the catalytic centre [41,42]. The RNB active site is buried at the bottom of narrow channel and can only be reached by single-stranded RNA at least 7 nt long [40]. DIS3 can unwind substrates with intra- or intermolecular secondary structures as long as there is an unstructured region of at least 4–5 nt at the 3' end of the RNA. The force of the active site pulling on the 3' end of dsRNA accumulates as elastic tension so that about every 4 nt the tension reaches a threshold value and is released in a “burst” to unwind 4 nt of the duplex at a time [43]. The endonucleolytic PIN active site consists of four acidic amino acids that coordinate two divalent metal cations and is thought to function in releasing natural exosome substrates that are stalled at sites of strong secondary structure. The PIN domain cannot cleave double-stranded RNA but circular and linear single-stranded RNA are both substrates [33].

**Figure 2:**
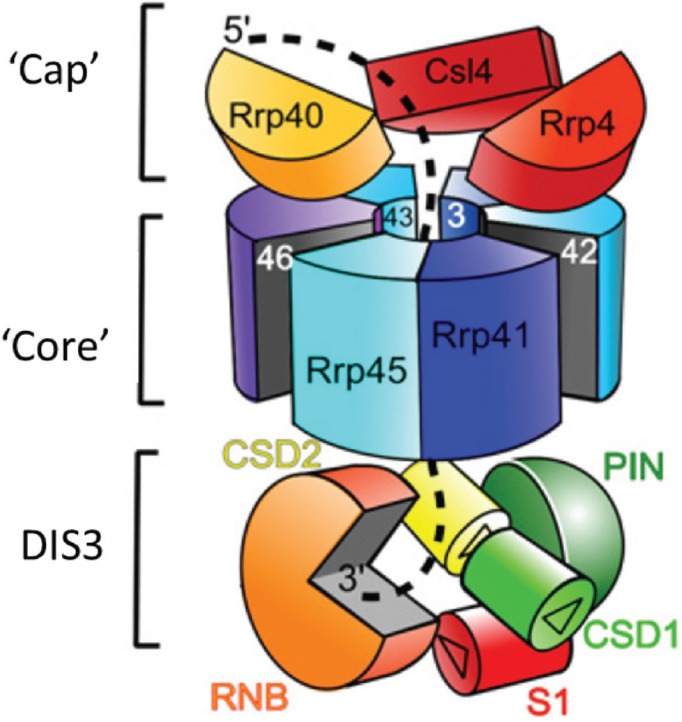
The exosome complex in association with DIS3. The inactive “core” exosome consists of nine subunits. Three subunits form an RNA binding “cap” structure (shades of red/orange). The remaining six subunits form a ring structure through which the RNA substrate is channeled (shades of blue). The exosome gains its activity by association with DIS3 at base of the ring structure. Adapted with permission from Luisi, B. *Structure* 2009, *17*, 1429–1431, doi:10.1016/j.str.2009.10.006 [46].

In mammals, DIS3 functions as one of the three catalytic subunits of the exosome, along with DIS3L and Rrp6, a distributive exoribonuclease which belongs to the RNase D family [5,44,45]. The exosome is a multi-protein complex composed of nine catalytically inert subunits that make up a two-layered barrel-like structure (Figure 2). The upper layer is composed of a “cap” of three S1 or KH domain RNA binding proteins, Rrp40, Csl4 and Rrp4 which rests on a “core” ring of six proteins, Rrp41–46, all with homology to RNase PH [46]. The recently solved crystal structure of the *S. cerevisiae* exosome complex shows Rrp44 (Dis3) to be anchored at the bottom of the exosome core through interactions with the PIN domain and CR3 motif [44,47]. Rrp6, can associate with the exosome cap, forming an 11-subunit complex. *In vitro* evidence suggests that DIS3 can act independently of the exosome although this has not been shown *in vivo* [5,45]. *In vitro*, when bound to the exosome, the activities of DIS3 and Rrp6 are suppressed through allosteric effects that diminish their RNA binding ability [5,48]. This may suggest that the exosome core modulates the RNase activities as part of a regulatory process that controls RNA decay.

The central channel of the exosome is only wide enough to accommodate single-stranded RNA, so secondary structures must be unwound from the cap by either the nuclear TRAMP complex or cytoplasmic Ski complex [46,49,50]. Substrates targeted to DIS3 can either enter the catalytic domain directly or be threaded through the central channel of the exosome to the exo- or endoribonuclease sites at the bottom. Recent data suggests that substrates for processing are targeted directly to DIS3, whereas some substrates for degradation must first be threaded through the exosome core [51,52]. Substrates targeted to Rrp6 are threaded through the central channel and divert off laterally beneath the S1/KH cap to access the Rrp6 active site. It is unknown how RNAs are differentially targeted to DIS3 or Rrp6; it appears stochastic but could be determined by additional factors *in vivo* [5]. Interestingly, Rrp6 appears to enhance the activity of DIS3 in the 11-subunit exosome complex [5] but the mechanism behind this is unknown. How the activities of these two enzymes cooperate *in vivo* is also unknown, however they are known to work sequentially in the maturation of 5.8 S rRNA [53].

**Figure 3:**
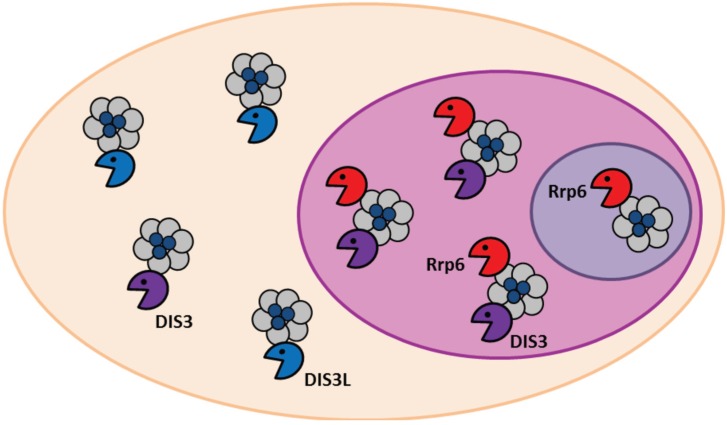
Sub-cellular localisation of the different human exosome complexes. The non-catalytic exosome core (grey) is present in the nucleus, cytoplasm and nucleolus but associates with different 3' to 5' catalytic subunits depending on the compartment. In the nucleus the exosome associates simultaneously with DIS3 (purple) and Rrp6 (red). In the cytoplasm the core associates with the cytoplasmic-restricted DIS3L (blue) and separately with DIS3 but in lower amounts. In the nucleolus the exosome binds only to Rrp6. It remains to be determined whether the exosome or the catalytic subunits exist on their own.

Subcellular compartmentalisation of exoribonucleases is an important control mechanism in the temporal and spatial regulation of RNA processing and decay. The subcellular localisation of DIS3 homologues and the different exosome subunits has not been investigated in great depth, besides two studies in *Drosophila* [45,54] and one study in human-derived HeLa and HEK-293 cells [31]. It is generally agreed that DIS3 is nuclear, excluded from the nucleolus with minor pools being found in the cytoplasm; whereas Rrp6 is found in both the nucleolus and nucleus and DIS3L is solely cytoplasmic (Figure 3). However, in some *Drosophila* S2 cells, Dis3 has shown restricted localisation to the cytoplasm and the localisation pattern differed from cell to cell [54]. Furthermore, a flag-tagged version of DIS3 expressed in a stable HEK-293 cell line showed only a nuclear localisation with no signal being detected in the cytoplasm [31]. The functional significance of these localisation patterns remains to be determined. DIS3, along with other exosome components, may localise to different regions of the cell depending on cell-cycle stage or changes in growth conditions. Interestingly, flag-tagged Dis3 co-localises with the nuclear lamina in *Drosophila* cells [54]. The importance of this nucleo-peripheral localisation is unknown; however DIS3 could be critical for surveillance during mRNA export. This hypothesis is supported by previous studies that show both *S. pombe* and human DIS3 interact with Ran, which is essential for nucleocytoplasmic transport of proteins and ribonucleoproteins [14,55].

The predominantly observed nuclear localisation of DIS3 is thought to be controlled by two nuclear localisation signals at the C-terminus of the protein [45]. DIS3 is known to target both nuclear and cytoplasmic RNAs but it is not known whether a distinct pool of DIS3 proteins exist in each compartment, or if a single, shuttling pool is responsible for the processing and/or turnover of targets in both the nucleus and cytoplasm. N-terminal domains also appear to contribute to DIS3 subcellular localisation but they do not contain nuclear localisation sequences [56]. N-terminal domains may contain an additional regulatory sequence or they may act by maintaining the proper structure of the enzyme, such that the C-terminal nuclear localisation signal is kept in a functional conformation.

## 3. Molecular Functions of DIS3

The continuous synthesis and degradation of RNAs allow the metabolic changes required for proper cellular function. In association with the exosome, DIS3 is the common effector of a vast range of RNA metabolic pathways functioning in mRNA quality control, regulation of gene expression and small RNA processing. Although not discussed here, the ability of the exosome to handle such a diversity of substrates is down to a network of auxiliary factors which interact with exosome to recruit it to particular substrates [57]. The following section aims to discuss the role of DIS3 and the exosome in mRNA decay as well as in small RNA processing and decay. The relative contributions of Rrp6 and Dis3 to the degradation of the many exosome substrates are still not fully understood, however where specificities are known this has been indicated.

### 3.1. Role of DIS3 in mRNA Decay

**Figure 4:**
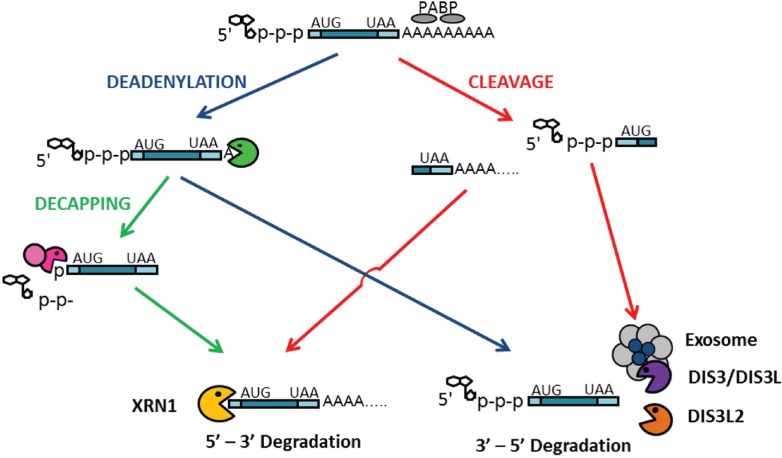
Overview of the mRNA degradation pathways in eukaryotes. Messenger RNAs first undergo removal of the 3' poly-A tail (deadenylation) allowing access for 3' → 5' degradation by the exosome complex and DIS3. Following deadenylation the mRNA can undergo removal of the 5' cap (decapping) exposing the mRNA to degradation by the 5' → 3' exoribonuclease XRN1. Alternatively mRNAs can undergo endonucleolytic cleavage (e.g., due to RNAi, or nonsense-mediated mRNA decay in some organisms) creating two fragments, each of which is susceptible to either XRN1 or the exosome and the DIS3 paralogues.

mRNA degradation in eukaryotes involves a number of complex and interconnected pathways that all converge on three common mechanisms. mRNAs must first be either deadenylated and de-capped or internally cleaved to allow access for either the exosome and paralogues of DIS3 or the 5'-3' exoribonuclease, XRN1 (Figure 4). Deadenylation removes the poly-A tail from transcripts to allow access for 3' to 5' degradation by DIS3 and the exosome [58]. The 5'-cap may then be removed by decapping enzymes, leaving the transcript vulnerable to 5' to 3' degradation by the XRN1 exoribonuclease [59]. Finally, transcripts can be endonucleolytically cleaved to create two fragments which are susceptible to degradation by either DIS3 and the exosome or XRN1. Many different pathways exist upstream of these processes to target particular substrates for degradation. These pathways can be divided into quality-control and regulated-decay pathways and are described in the following three sub-sections.

#### 3.1.1. Role of DIS3 in RNA Quality-Control Pathways

Aberrant and faulty transcripts must be detected by the cell to prevent the production of potentially toxic protein products. Surveillance mechanisms exist both in the nucleus and in the cytoplasm to detect errors at all stages of mRNA production and maturation. In the nucleus mRNAs that are faulty due to errors in transcription, export or processing are degraded. Both the 3' to 5' and 5' to 3' pathways are involved in nuclear mRNA turnover but which is used depends on the substrate. The exosome is known to specifically degrade un-spliced pre-mRNAs [6] and mRNAs with defective poly-adenylation [7]. Interestingly, there is evidence that pre-mRNA surveillance by the exosome takes place during transcription. The interaction and co-localisation with the elongation factor, Spt6, and RNA polymerase II, in *Drosophila*, suggests the exosome may function co-transcriptionally *in vivo* as part of a checkpoint that monitors proper pre-mRNA processing [60].

Surveillance pathways that take place in the cytoplasm are translation dependent and include nonsense mediated decay [61], non-stop decay [62,63] and no-go decay [64]. Nonsense mediated decay (NMD) is triggered by transcripts that contain a premature termination codon (PTC). In NMD a sequence of protein binding events are triggered [65] which subsequently leads to the decay of the transcript by either the 5' to 3' or 3' to 5' pathway [61]. Although not yet clear, evidence suggests that Rrp6 is predominant over DIS3 in targeting these NMD substrates [66]. Non-stop decay targets mRNAs that lack a stop-codon and as a consequence translation continues along the poly-A tail. In yeast and mammalian cells, a stalled ribosome at the 3' end of a transcript is detected and bound by Ski7 which recruits the Ski complex and the exosome to deadenylate and degrade the transcript [62].

The most recently discovered RNA surveillance pathway, no-go decay, prevents translation of transcripts with a strong secondary structure. Ribosomes stalled by the secondary structure are detected and the mRNA is endonucleolytically cleaved. The endonuclease responsible has not been identified but seems to require Dom34 and HSB1, proteins which are related to the eukaryotic translation release factors, eRF1 and eRF3 [64]. Once the transcript has been cleaved into two fragments, it is degraded by either the exosome or XRN1.

#### 3.1.2. Role of DIS3 in Regulated mRNA-Decay Pathways

Alternative to the degradation of aberrant mRNAs, mRNAs may be subjected to regulated degradation as a means of controlling gene expression. This can occur through cis-encoded destabilising elements in the 3' UTRs or by the RNA-induced silencing complex (RISC).

Two types of cis-encoded destabilising elements exist: AU-rich elements (ARE) and GU-rich elements (GRE), both found in the 3' UTRs of a number of mRNAs. AREs are found in short-lived mRNAs coding for proteins that mediate regulatory responses in the cell, such as inflammatory or stress responses (e.g., GM-CSF, c-fos, and cmyc) [67]. AREs exert their effect on post-transcriptional gene expression by recruiting trans factors. These ARE-binding proteins (AUBPs) can promote transcript degradation by recruitment of the CCR4-NOT complex resulting in deadenylation of the mRNA and subsequent degradation by DIS3 and the exosome [8,9]. The function and abundance of GREs is less understood but they have been shown to regulate a different repertoire of genes and have a more modest effect on mRNA stability [68].

MiRNA-mediated degradation of mRNAs provides another means of modulating gene expression. miRNAs are short RNA molecules which generally act to downregulate target expression by either repressing translation or causing degradation of their target mRNA by the RNA-decay machinery. In plant cells, miRNAs typically base-pair with their targets with almost perfect complementarity which results in cleavage of the target mRNA by RISC (RNA-induced silencing complex) and subsequent degradation of the 3' section by AtXRN4. The 5' section is thought to be degraded by the exosome [69]. In animal cells, the molecular mechanisms of miRNA-mediated gene silencing are still not clear, probably due to the existence of such a huge diversity of mechanisms. However, in most cases miRNAs are usually only partially complementary to their targets and direct endonucleolytic cleavage of targets rarely occurs. Although it was previously thought that the levels of miRNA-targeted mRNAs remained unchanged, recent evidence suggests that mRNA degradation rather than translational repression is the main mechanism of silencing [69]. For these targets, the CCR4-NOT complex is recruited resulting in deadenylation of the mRNA targets which are then decapped and degraded 5' to 3' by XRN1 [70]. However, there is currently no evidence to suggest DIS3 or the exosome are involved in the degradation of miRNA-repressed mRNAs in animal cells.

### 3.2. Role of DIS3 in Small-RNA Processing and Decay

Not all RNA substrates that are targeted by the RNA decay machinery are destined for complete degradation. DIS3 and the exosome were originally discovered in yeast to be involved in the processing of ribosomal RNAs (rRNAs) [11] and were only later discovered to have a function in mRNA surveillance. rRNA, small nucleolar RNA (snoRNA), small nuclear RNA (snRNA) and tRNA species are all transcribed as pre-RNAs, which must then be cleaved and/or trimmed to produce functional small RNA products [71]. The exosome is generally responsible for processing these stable nuclear RNAs by trimming the extended 3' ends of primary transcripts down to their mature length.

For example, rRNA synthesis in yeast begins with the synthesis of a 35S precursor-rRNA in the nucleolus. The pre-rRNA gets internally cleaved in a series of steps to produce a number of smaller fragments including a 7S intermediate. In the nucleus, the Dis3-exosome complex is required for the 3' end processing of the 7S intermediate into the mature 5.8 S rRNA and for the degradation of the 5' external transcribed spacer removed from the full length pre-rRNA transcript [72]. Final maturation of 5.8 S rRNA takes place in the cytoplasm where it undergoes exonucleolytic processing at the 3' end, also by the exosome. Additionally, snoRNAs and snRNAs which participate in rRNA processing and modification and pre-mRNA splicing respectively, are excised from polycistronic precursors or from mRNA introns and undergo multi-step 3' processing that involves the nuclear exosome [10].

Dis3 has been found to specifically degrade tRNA species in yeast. This function was first discovered in *S. cerevisiae* tRNA methyltransferase mutants that produce hypomodified tRNAs [73,74]. These tRNAs lack a single modification which may subtly affect their folding but otherwise are mature and functional. The intact exosome lacking only the catalytic activity of Dis3 fails to degrade the hypomodified tRNA, showing this to be a specific Dis3 substrate. Additionally, in yeast Dis3 mutants, both mature and precursor tRNAs are markedly increased [75]. This phenotype is intensified in Dis3 exo-endo-double mutants suggesting PIN activity contributes significantly to tRNA degradation, as expected from highly structured substrates. Interestingly, this study revealed that more than 50% of tRNAs that are transcribed are degraded by Dis3 and never reach the functional pool of mature tRNAs in wild-type cells.

As mentioned previously, miRNA-mediated degradation of mRNAs is an important means of modulating gene expression. The unique combination of miRNAs contributes to a cell’s specific array of protein expression and their misexpression is associated with many types of human cancer [76]. For this reason, miRNA production is itself subject to several levels of regulation [77]. As well as transcriptional regulation, post-transcriptional regulation through RNA degradation is also important. This can occur indirectly through the regulation of RNA-binding proteins such as the cleavage of DCGR8 mRNA by Drosha, leading to its degradation [78,79], or directly by targeting either the pri, pre- or mature miRNA.

Many miRNAs that are known to be degraded in different organisms have as yet undefined nucleases. Nevertheless, the exosome and sometimes DIS3 specifically have been found to be involved in the turnover of several miRNAs. In *Drosophila* wing imaginal discs, Dis3 knock-down has been found to increase the expression of the mature form of *miR-252-5p* but not the precursor, suggesting Dis3 may be functioning to specifically degrade the mature miRNA as a means of regulation. Another miRNA, *miR-982-5p*, decreases in expression in Dis3 knock-down discs, suggesting Dis3 may be involved in processing the precursor miRNA into its mature form [80]. Also in *Drosophila*, a family of miRNAs have been discovered that are encoded in introns, which are processed in an exosome mediated biogenesis pathway. These mirtrons bypass normal Drosha cleavage and are processed into pre-miRNAs by the spliceosome. After splicing the 3' tail is trimmed by the exosome [81]. Additionally, DIS3 and Rrp6 have been found to be involved in the degradation of pre-miRNAs in mammalian cells. Unlike Rrp6, knockdown of DIS3 does not seem to affect the level of mature miRNAs but does cause an increase in several truncated pre-miRNAs, suggesting DIS3 is involved in the quality control of pre-miRNAs [82]. Interestingly, this study found the activity of DIS3 on pre-miRNAs to be stimulated by uridine tails, which stimulate the uridyl transferases TUT4 and TUT7, providing a positive feedback-loop in the degradation of Ago-bound pre-miRNAs.

One of the major classes of nuclear exosome substrates in humans is PROMoter uPstream Transcripts (PROMPTs). Similar to cryptic unstable transcripts (CUTs) in yeast, PROMPTS are short-lived RNA species, between 200 and 600 nucleotides in length, transcribed upstream of the promoters of active protein-coding genes. PROMPTs are transcribed by any of the three RNA polymerases and have 3' poly-A tails as well as 5' cap structures. Evidence suggests that most, if not all, actively transcribed RNA pol II genes have associated PROMPTs, but they seem to be especially prominent at TATA-less, CpG-rich promoters with broad transcription start site (TSS) regions [83]. PROMPTs tend to be generated between 500 and 2500 nt upstream of the TSS and although not linked with TSS-associated RNAs (formed by RNA Pol II backtracking and stalling), their transcription is positively correlated with downstream gene activity. PROMPTs are currently poorly understood but could function in regulating the expression of downstream genes by providing reservoirs of RNAPII which facilitates rapid activation of the downstream gene [84]. PROMPTs are only detected when exosome subunits are depleted. Single knock-downs of DIS3 or Rrp6 yield a much lower stabilisation than double depletion of both catalytic subunits [31,83], however human DIS3 mutant cells show a significant stabilisation of PROMPTs without simultaneous mutation of Rrp6 [85].

## 4. Biological Functions of DIS3

Although its role in RNA metabolism is well-documented, the biological functions of DIS3 responsible for the observed phenotypes in mutants are less well known. There are a number of studies in yeast and *Drosophila* which pertain to the biological activity of DIS3 (Table 1), however sufficient functional studies of DIS3 in human cells do not exist. Nevertheless, this protein is strikingly conserved across eukaryotes meaning studies in lower organisms may yield useful insight into its function in human cells.

### 4.1. Role of DIS3 in Cell-Cycle Regulation

There are a number of studies which present evidence for a role of DIS3 in regulation of the cell-cycle. Dis3 was first discovered in a mutant fission yeast strain to cause non-separation of sister chromatids during anaphase [12,13]. Subsequently, the *S. pombe* Dis3 homologue was found to bind to the human GTPase Ran, a member of the RAS superfamily [14] which functions in spindle assembly and the regulation of cell cycle progression as well as in nucleocytoplasmic transport [86,87]. RanGTP specifically functions to activate spindle assembly factors by releasing them from complexes with importins [88,89]. At kinetochores, increased Ran-GTP levels displace some spindle assembly checkpoint (SAC) components to allow activation of the anaphase-promoting complex (APC) [90], facilitating cell-cycle progression. Interestingly, the same RNA processing phenotype has been observed in both Dis3 and Ran yeast mutants, suggesting that Ran may regulate the assembly or disassembly of Dis3 and the nuclear exosome [91].

More recently, *S. pombe* Dis3 mutants have been shown to have elongated metaphase spindles and a block in metaphase to anaphase transition [16]. Like Ran, Dis3 appears to be required for correct kinetochore formation and function. The kinetochore consists of many proteins whose functions include anchoring of chromosomes to the mitotic spindle, verification of anchoring, activation of the spindle checkpoint and participation in force generation to propel chromosome movement during cell division [92]. Kinetochore formation is monitored by the spindle checkpoint protein Mad2. In single Dis3 mutants, Mad2 restrains mitotic progression but in Dis3 Mad2 double mutants, cells proceed to anaphase without proper chromosome segregation, generating aneuploid cells.

**Table 1:** List of phenotypes observed in DIS3/exosome mutants and knock-downs in various organisms. Where applicable the corresponding amino acid (AA) in human DIS3 has been given, along with the affected domain.

Phenotype/Process Affected	Organism/Cell Line	Knock-Down or Mutant (AA Position)	Corresponding Human DIS3 AA Position	Conserved in Humans?	Domain	DIS3/Exosome Subunit Referred to as	Refs.
Non-separation of sister chromatids	*S. pombe*	P509L	P509—Conserved (based on predicted *S. pombe* sequence)	Yes	RNB	dis3	[12,13]
Mitotic control and interaction with Ran GTPase	*S. cerevisiae*	G562D, E565K, V566G	G562 E565 V566	Yes, all three	RNB	Dis3sc	[14,91]
Aneuploidy, spindle assembly, metaphase to anaphase transition and kinetochore function	*S. pombe*	P509L	P509—Conserved (based on predicted *S. pombe* sequence)	Yes	RNB	dis3	[16]
Cell-cycle regulation and microtubule production	*S. cerevisiae* *D. melanogaster*	G562D, E565K, V566G Knock-down	G562 E565 V566	YesN/A	RNBN/A	Dis3	[14,66,93]
Larval lethality, no wings	*D. melanogaster*	Knock-down	N/A	N/A	N/A	Dis3	[80]
Centromeric transcript turnover and heterochromatin silencing	*S. pombe*, *S. cerevisiae*	P509L	P509	Yes	RNB	Dis3/Rrp44/Rrp4	[16,94,95]
Antibody diversification	*M. musculus CH12F3 lymphoma cells, Human Ramos B lymphoma cells, HEK-293 cells*	Knock-down	N/A	N/A	N/A	Dis3/Rrp44/Rrp40, exosome subunit	[17]

**Figure 5:**
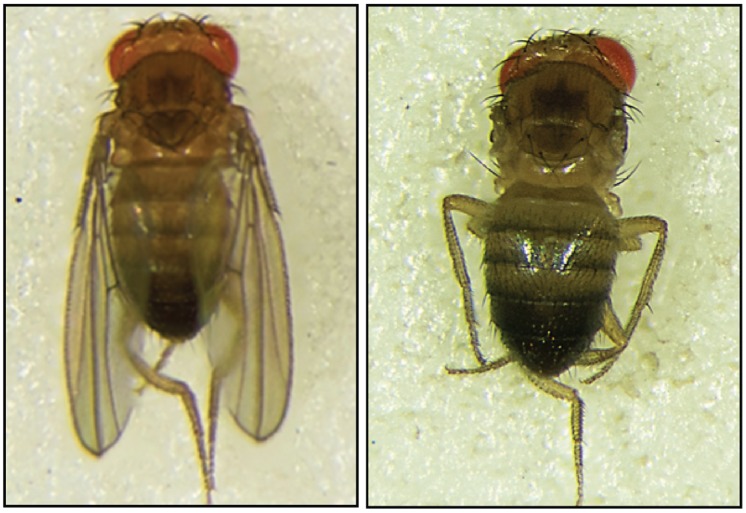
Knockdown of Dis3 in *D. melanogaster* wing imaginal disc results in a severe “no wing” developmental phenotype (right) compared to wild-type (left) [80].

Andrulis *et al.* provide further evidence that DIS3 is involved in mitotic progression as perturbation of Dis3 in *S. cerevisiae* affects microtubule localization and structure [15]. RNA-seq analysis showed broad changes in the levels of cell cycle and microtubule related transcripts in Dis3 mutant strains. Similar work in *Drosophila* S2 cells showed that the knock-down of Dis3 also predominantly affects the expression of cell cycle-related transcripts [66]. Another study using transgenic flies showed ubiquitous loss of Dis3 to cause larval lethality. In the same study, a spatial knock-down of Dis3 only in the wing pouch region of the imaginal disc was performed, yielding flies with a severe “no wing” phenotype [80] (Figure 5), revealing the essential role of this protein in development.

Although previous siRNA-based experiments in human cells did not show an effect of DIS3 knock-down on growth rate, Tomecki *et al.* subsequently showed a mutation-specific effect on the growth of HEK-293 cells. Cell lines were created that expressed inducible exogenous variants of DIS3 with multiple myeloma associated mutations. Cells expressing DIS3 variants with D487N or R780K substitutions proliferated at a slower rate compared with the wild-type cell line. Additionally, when the endonucleolytic PIN domain is mutated alongside mutations in the RNB domain, synthetic lethality and a higher accumulation of PROMPTs are observed, suggesting the two catalytic domains cooperate to degrade substrates. The same group have shown homozygous conditional knock-outs of human DIS3 from the DT40 Cre1 cell line is lethal [85].

What is the mechanism by which DIS3 is affecting mitosis? One suggestion is that DIS3 could be processing a gene needed for kinetochore formation [16]. Another, supported by recent data, is that DIS3 has a link with heterochromatin silencing at the centromere [16,94,95]. Previously, dense chromatin packaging in heterochromatic regions was thought to inhibit transcription leading to low level gene activity [96]. However, recent evidence from budding and fission yeasts suggests that rapid nuclear turnover of heterochromatic transcripts, reinforces transcriptional silencing [97]. The deletion of Dis3 considerably increases levels of transcripts from silent centromeric and telomeric loci [20,86,95]. As the centromere is essential for proper segregation of chromosomes, which is disrupted in Dis3 mutants, centromeric heterochromatin silencing represents a plausible role for DIS3 *in vivo*.

### 4.2. Role of DIS3 in Generating Antibody Diversity

Interestingly, the exosome has also been implicated in recruiting activation-induced cytidine deaminase (AID) to chromatin in mammalian B-cells, where DIS3 may be functioning specifically in degrading nascent RNA during the DNA repair process [17]. AID functions by converting methylated and 5-hydroxymethylated cytidine residues into uracil and thymine respectively, which are subsequently recognised by the DNA repair machinery and converted into double-strand breaks (DSBs). The double strand breaks are mostly repaired locally between IgH regions as part of two immunoglobulin gene diversification processes: Somatic hypermutation (SHM) and IgH class switch recombination (CSR). Since AID can only act on single-stranded DNA (ssDNA), these processes only occur during gene transcription when the DNA duplex is opened up. However, although able to access the non-template strand directly, AID has no known activity on RNA/DNA hybrids; therefore the mechanism by which it accesses the template strand which is hybridised to the nascent transcript, is unknown.

*In vivo* knockdown, ChIP, and physical association studies by Basu *et al.* provide evidence that the RNA exosome functions in recruiting AID to both strands of transcribed duplex DNA substrates [17]. To do this, the exosome along with one of its catalytic subunits, must remove the template RNA. As discussed previously, the exosome has been shown to interact with elongating RNA polymerase II [60]. However, it does not engage RNA substrates that lack a free single-stranded 3' end and RNA still attached to RNA polymerase only has a free 5' end. Therefore Basu *et al* propose a model whereby the exosome functions on stalled Pol II units that backtrack to reveal a free 3' end [17]. DIS3 or Rrp6, may function in degrading the nascent RNA in the 3' to 5' direction, leaving the template strand as ssDNA substrate for AID. Interestingly, in the event of DSB repair failure after AID-directed deamination, chromosomal translocations can result, as is often observed in multiple myeloma.

## 5. DIS3 and Disease

### 5.1. DIS3 and Cancer

The earliest study linking DIS3 to cancer identified it as a metastasis-related gene. Two independent gene expression profiling studies of colorectal cell lines and human tissues identified overexpression of DIS3 as high as 38-fold in primary and metastatic tumours relative to normal colonic mucosa [18,19]. Another study has shown a significant overexpression of DIS3 in colorectal carcinomas compared to adenomas [20,98]. This observed overexpression could be due to an amplification of the DIS3 locus, 13q22, frequently observed in colorectal cancer. Conversely, the DIS3 locus is often deleted in chronic lymphocytic leukaemia (CLL) and patients have been found to display loss-of-heterozygosity (LOH). Sequenced germline DNA from five families with CLL showed five amino acid changes within DIS3. DIS3 was also shown to be under-expressed 2.8-fold in a CLL leukaemic clone compared with normal B-cells [22], suggesting decreased expression is a consequence of the decrease in copy number. This difference in copy number between colorectal cancer and CLL may suggest a tissue-context dependent role of DIS3 in promoting cancer development. DIS3 has also appeared in linkage studies of breast cancer patients [21]. However the significance of this is unclear as it involves polymorphisms rather than deleterious mutations.

DIS3 may also be biologically relevant in melanoma. In superficial spreading melanoma (SSM) cells, DIS3 has reduced expression compared to normal melanocytes because one chromosomal copy is deleted. In contrast, DIS3 is overexpressed in nodular melanoma (NM) cells [23]. Furthermore, SSM cells display sensitivity to mebendazole, a microtubule-destabilizing drug, whereas NM cells are resistant. This is consistent with the function of DIS3 in the regulation of chromosomal segregation during mitosis (see Section 4.1) [16]. SSM and NM are believed to represent sequential phases of linear progression from radial to vertical growth, yet recurrent differential deletions such as that of DIS3 suggest SSM and NM might be the result of an independent pathway. However, a recent meta-analysis with combined experimental validation of five microarray-based melanoma datasets did not identify DIS3 to be part of a biomarker signature for melanoma [24].

Whole genome sequencing studies have identified missense mutations in DIS3 to occur in ~4% (4/106) of Acute Myeloid Leukaemia (AML) patients [99]. In all affected patients, mutations mapped to the exoribonucleolytic RNB domain. AML develops as a clonal evolution of haematopoietic progenitor cells (HPSC/blasts). A HPSC acquires an initiating event which increases its proliferation and genetic instability, causing the clone to expand. Many subclones evolve from the founding clone leading to an oligoclonal malignant tumour [100]. Alleles found to have a variant allele frequency (VAF) of 50% usually represent heterozygous somatic mutations that are present in all cells within the sample. DIS3 is mutated in both primary tumour and relapse samples at a VAF between 37% and 47%, suggesting a heterozygous event in these cases. However, whether DIS3 mutations initiate clonal expansion of the HSPC or cooperate to give the clone an additional advantage is still unclear [99].

### 5.2. DIS3 and Multiple Myeloma

The most striking association between DIS3 and cancer is probably the finding that DIS3 is recurrently mutated in multiple myeloma (MM). Multiple myeloma is defined by a malignant proliferation of monoclonal antibody (also called M protein)-secreting plasma cells and counts for 20% of deaths related to haematological malignancies [101,102]. MM begins as an asymptomatic pre-malignant syndrome of clonal plasma cell proliferation termed monoclonal gammopathy of undetermined significance (MGUS) and eventually evolves into plasma cell leukaemia, an aggressive extramedullary disease. Multiple myeloma is a genetically heterogenous disease where different patients fall into distinct genetic subgroups that determine different clinical outcomes.

**Figure 6:**
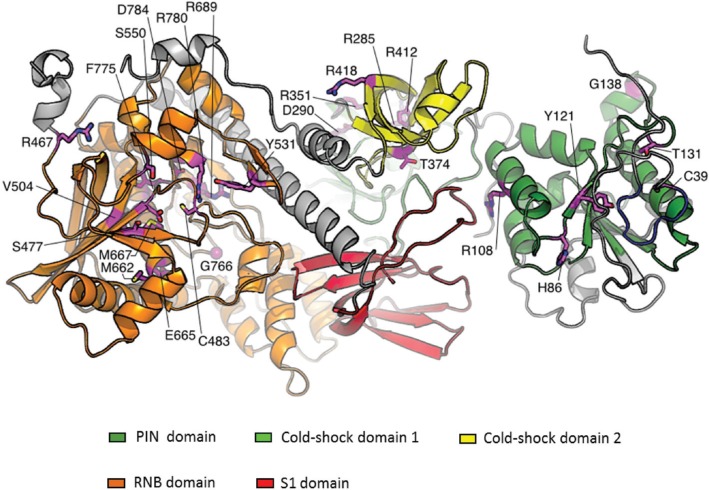
Three-dimensional model of DIS3 illustrating the position of amino acids substituted by myeloma-associated mutations. Mutations in pink. DIS3 domain functions—RNB domain: Exoribonucleolytic; PIN domain: Endoribonucleolytic; CSD1 and CSD2: Cold-shock; S1: RNA binding. Modelled according to the recently solved *S. cerevisiae* Rrp44 structure [44] using Phyre2 [103] and the webserver “Site Directed Mutator”.

To date 34 of 306 (11%) myeloma patients analysed by whole genome or exome sequencing studies have been found to have missense mutations in DIS3 that may be functionally relevant (Figure 6) [25,26,27,28]. In most patients DIS3 mutations correlate with deletions of 13q and a few patients were also found to be associated with copy neutral loss of heterozygosity (cnLOH) that results in the presence of homozygous DIS3 mutations. A recent amplicon sequencing study identified three hotspot mutations (R780, D488 and E665) within the RNB domain of DIS3 and investigations in HEK-293 cells indicate that the R780K mutation leads to a lower proliferation rate compared to the WT cell line, suggesting a loss-of-function phenotype which would classify DIS3 as a tumour-suppressor gene. Moreover, biochemical assays performed using recombinant versions of DIS3 bearing MM-associated mutations indicate that in the majority of cases, these mutations abolish DIS3 exoribonucleolytic activity [85]. Analyses using available structural information and predictive tools also suggest that most myeloma mutations have a destabilising effect on the enzyme. For example, R780K, found in six multiple myeloma patients, involves an amino acid which is directly involved in binding to the phosphate backbone of the incoming substrate so is highly likely to affect catalysis. Also, S477R, found in another patient, is a drastic mutation from a small to very large amino acid, with a charge reversal. It is next to a loop that contains residues involved in magnesium ion coordination and is therefore also predicted to have an impact on the catalytic activity of DIS3.

Similar to many other cancers, multiple myeloma has been found to develop as a consequence of a clonal evolution of cells [104,105,106]. In multiple myeloma specifically, the initial immortalisation of the cell usually occurs by the acquisition of a chromosomal abnormality [107]. Chromosomal abnormalities can be classified into hyperdiploid (trisomies) or non-hyperdiploid subtypes. Curiously, DIS3 mutations are most commonly seen in non-hyperdiploid subtypes [25,26,27]. Non-hyperdiploidy involves translocations of the IgH locus with other chromosomes and is caused by aberrant class-switch recombination (CSR) in B-cells. Aberrant CSR brings oncogenes under the influence of the IgH enhancer region leading to their up-regulation. These primary genetic events co-operate with secondary lesions to produce the founding clone of myeloma [108]. Many subclones evolve from the founding clone, leading to an oligoclonal malignant tumour. This process of clonal evolution creates extensive heterogeneity not only between patients but also within individual cases. Moreover, intra-patient clonal heterogeneity can change over time as a result of treatments that incompletely suppress the whole tumour population, leading to the emergence of an aggressive minor subclone. Recently there has been a move towards using combinatory chemotherapy in an attempt to eradicate all clones as well as avoiding selection of minor aggressive ones [109].

High throughput studies have provided semi-quantitative analysis of the size of the clonal populations carrying a particular mutation within an individual tumour. It was anticipated that mutations arising in all the clones would take part in initiating myeloma whereas mutations present only in some subclones would be potentiators of the disease. However, it appears that the situation is not quite that simple. Mutations in DIS3 were found to be both clonal in some patients and sub-clonal in others meaning they are functioning sometimes as the former and sometimes as latter [25,28]. This observation is not restricted to DIS3 but rather applies to the other ten significantly mutated genes in myeloma patients, KRAS, NRAS, TP53, FAM46C, BRAF, TRAF3, PRDM1, CYLD, RB1 and ACTG1.

Within patient samples, some patterns of cooperation and exclusion can be identified between mutations in DIS3 and other genes. DIS3 mutations seem to be mutually exclusive with mutations in FAM46C. Collectively, DIS3 and FAM46C mutations are observed in 21% of patients [25]. The precise function of FAM46C is yet unknown, however there is evidence it belongs to a family of nucleotidyltransferases [110] and it was recently shown to function as an mRNA stability factor that interacts with poly-A-binding protein cytoplasmic 1(PABPC1) and binds to CU rich motifs within the 3' UTRs of some mRNAs [111]. In support of this function, Chapman *et al*. have found its expression to be highly correlated to the expression of a set of ribosomal proteins and translation initiation factors [30]. DIS3 is known to function in the maturation of rRNA, suggesting these two genes could be involved in the same cellular pathway.

Conversely, DIS3 mutations mostly seem to occur in parallel with a hemizygous (monallelic) deletion of the RB1 region (13q14), either as del (13q) or as an interstitial deletion of the RB1 locus. The gene of interest at 13q14 may be RB1 (retinoblastoma tumour suppressor protein), or one of the miRNAs at this locus which are under-expressed in CLL and MM (*miR-15a/16*). This raises the possibility that mutation and selection of DIS3 as a driver mutation in myeloma is dependent on deletion of 13q14. However, more NGS studies are needed to increase the sample size in order determine whether this correlation is significant.

Although there is very little data on the clinical impact of DIS3 mutations, one very recent study has identified a trend towards a shorter median overall survival for patients with DIS3 mutations. Patients carrying DIS3 mutations in minor subclones of their tumours also showed a significantly worse response to therapy compared to patients with DIS3 mutations in the major subclone [28]. Nevertheless, minor subclones tend to accumulate 17p deletions which may also explain this trend.

While the cohort sizes in these genome-wide sequencing studies are large, the absolute number of patients with DIS3 mutations is nonetheless relatively small. For this reason, more patient sequencing studies need to be conducted in order to fully determine the clinical implications of DIS3 mutations to myeloma prognosis.

## 6. Conclusions and Future Prospects

DIS3 has been proven to be far more than simply an RNA disposal machine, with essential roles in both gene regulation and small RNA processing. The phenotypes of DIS3 knockdowns and mutants have led to both mechanistic and biological insights about the roles of this enzyme in the cell. Mutants often reveal specific developmental phenotypes, suggesting DIS3 may be targeting particular functional subsets of RNAs such as those involved in cell-cycle regulation, spindle assembly and kinetochore function. While a large body of structural and biochemical data exists, it is only in recent years that the functional diversity of DIS3 has been fully appreciated and DIS3 has been connected with human disease.

Although DIS3 has been found to be associated with multiple types of cancer including colorectal, melanoma and three types of blood cancers, there is no consensus about whether DIS3 is a tumour suppressor or oncogene. It seems to be over-expressed in some cancers and under-expressed in others, whilst displaying loss-of-function mutations in AML, suggesting the role of DIS3 might differ within the context of different tissues and tumourigenic pressures. However, the most significant association of DIS3 and human disease is the recurrent loss-of-function mutations in multiple myeloma patients. Although it is an extremely heterogenous disease, multiple myeloma is currently, for the most part, treated as a single entity with the result that therapeutic success is varied amongst individual patients. As the characterisation of the PML-RARA fusion showed, a lot of progress can be made by the identification of a single molecular event regarding disease definition. Therefore, identifying the role of DIS3 in oncogenesis may help to develop new targeted therapies for affected patients.

The mutant and knock-down phenotypes we have discussed in lower organisms and cell lines have the potential to yield insight into the role of DIS3 in myelomagenesis. The observations that DIS3 causes cell cycle arrest in many mutants may at first appear illogical in relation to its role in MM as a tumour suppressor gene. If DIS3 plays an important role in myeloma progression, we may expect DIS3 mutants to display over-proliferation or de-differentiation, phenotypes more consistent with those observed in tumours. However, when we consider that cancers are a result of a clonal evolution of cells that have acquired not just one, but many cooperating mutations, it is not surprising that mutation of just one of these genes in a model organism produces a different phenotype. This is demonstrated in single yeast Dis3 mutants that display elongated metaphase spindles. The production of faulty spindles leads to a block in metaphase to anaphase transition. In these single mutants, the intact Mad2 protein restrains mitotic progression because the chromosomes cannot be segregated properly, leading to eventual apoptosis. However, in Dis3 Mad2 double mutants, cells proceed to anaphase despite having elongated spindles meaning chromosomes are not properly segregated, leading to the production of aneuploid cells. This phenotype may have relevance to the pathogenesis of multiple myeloma of which 90% of patients display aneuploidy [112]. A combination of DIS3 and spindle-assembly checkpoint defects [113] may allow cells to progress through the cell-cycle without proper chromosome segregation generating aneuploidy cells which lead to the development of myeloma.

Another scenario by which DIS3 could be contributing to the development of myeloma is through the interaction between the RNA exosome and AID during the process of class switching and hypermutation in B-cells. Multiple myeloma is a malignancy of mature antibody-producing B-cells. DIS3 mutations could indirectly, through disruption of an interaction with AID, cause mistargeting of somatic hypermutation leading to chromosomal translocations. This fits with DIS3 mutations being almost exclusively observed in non-hyperdiploid subtypes *i.e*., those defined by IgH translocations [29]. Exactly how DIS3 mutations could contribute to this though remains to be elucidated. Alternatively, loss-of-function of DIS3 may either allow increased expression of other mutated genes, or may cause the overabundance of unstable noncoding transcripts such as PROMPTs (see previous discussion) that may indirectly affect expression of genes important for carcinogenesis.

The preferred method by which to experimentally verify these hypotheses is to use true myeloma models. The cell line OPM2 for instance, carries a DIS3 mutation in the PIN domain paired with cnLOH [29]; however, whether this mutation affects the exoribonucleolytic function of the enzyme remains to be investigated. Remarkably, there are not yet any reports on the specific biological functions of DIS3 in the mouse or in zebrafish. Further work on the biological role of this enzyme in model organisms is likely to shed light on its role in human cancers.

As well as performing functional assays to elucidate the role of DIS3, important information can be derived from mutational patterns revealed by genome-wide sequencing studies. Many mutations arising in myeloma do not co-occur in the same cell progeny suggesting disruption of one gene in a pathway may be sufficient to drive oncogenesis. As discussed, mutations in DIS3 and mutations in genes involved in protein translation and/or homeostasis such as FAM46C appear to be mutually exclusive in MM [26]. Conversely, DIS3 mutations mostly seem to occur in parallel with a hemizygous (monoallelic) deletion of the RB1 region (13q14) [26,27,28,29]. Identifying a pattern of cooperation or exclusion between DIS3 and other recurrent mutations may help us to understand its role in the pathogenesis of multiple myeloma.

It is useful to note that there appears to be an accumulation of mutations in the RNB domain of DIS3 but not DIS3L in myeloma, suggesting that inhibition specifically of the exonucleolytic activity of the nuclear exosome is what may facilitate oncogenesis. If this is the case, one might ask the question: Why Rrp6, which can act redundantly with DIS3 in the nucleus, does not compensate for the loss of DIS3 activity? This may be explained by a study in yeast which shows that when DIS3 is mutated, Rrp6 in the 11-subunit exosome complex is inhibited [57]. This dominant negative effect is thought to be caused by a blockage of the central channel by the ineffective interaction of RNA with the RNB domain. This may be an important consideration when performing knock-down experiments to investigate the role of DIS3 in myeloma development, as DIS3 knock-down is likely to have a different effect within the cell than DIS3 mutations, which probably affect the function of the protein. DIS3 mutant organisms rather than knock-down models are likely to provide a better insight into the role of DIS3 in myeloma pathogenesis.
